# A poor appetite or ability to eat and its association with physical function amongst community-dwelling older adults: age, gene/environment susceptibility-Reykjavik study

**DOI:** 10.1007/s10433-020-00588-1

**Published:** 2020-11-06

**Authors:** Milan Chang, Olof G. Geirsdottir, Lenore J. Launer, Vilmundur Gudnasson, Marjolein Visser, Ingibjorg Gunnarsdottir

**Affiliations:** 1 The Icelandic Gerontological Research Center, Landspitali University Hospital and University of Iceland, Reykjavík, Iceland; 2 Sport Science, School of Science and Engineering, Reykjavik University, Reykjavík, Iceland; 3 Faculty of Food Science and Nutrition, School of Health Science, University of Iceland, Reykjavík, Iceland; 4 Unit for Nutrition Research, University of Iceland and Landspitali University Hospital, Reykjavík, Iceland; 5 Epidemiology and Pop Science Lab, National Institute on Aging, National Institute of Health, Bethesda, MD USA; 6 Icelandic Heart Association, Kopavogur, Reykjavík, Iceland; 7 Faculty of Medicine, School of Health Science, University of Iceland, Reykjavík, Iceland; 8 Department of Health Sciences, Faculty of Science, Amsterdam Public Health Research Institute, Vrije Universiteit Amsterdam, Amsterdam, The Netherlands

**Keywords:** Ageing, Appetite, Body composition, Muscle strength, Physical function

## Abstract

A poor appetite or ability to eat and its association with physical function have not been explored considerably amongst community-dwelling older adults. The current study examined whether having an illness or physical condition affecting one’s appetite or ability to eat is associated with body composition, muscle strength, or physical function amongst community-dwelling older adults. This is a secondary analysis of cross-sectional data from the age, gene/environment susceptibility-Reykjavik study (*n* = 5764). Illnesses or physical conditions affecting one’s appetite or ability to eat, activities of daily living, current level of physical activity, and smoking habits were assessed with a questionnaire. Fat mass, fat-free mass, body mass index, knee extension strength, and grip strength were measured, and the 6-m walk test and timed up-and-go test were administered. Individuals who reported illnesses or physical conditions affecting their appetite or ability to eat were considered to have a poor appetite. The associations of appetite or the ability to eat with body composition and physical function were analysed with stepwise linear regression models. A total of 804 (14%) individuals reported having conditions affecting their appetite or ability to eat and had a significantly lower fat-free mass and body mass index, less grip strength, and poorer physical function than did those without any conditions affecting their appetite or ability to eat. Although the factors reported to affect one’s appetite or ability to eat are seldom considered severe, their strong associations with physical function suggest that any condition affecting one’s appetite or ability to eat requires attention.

## Introduction

Ageing is associated with a decline in physical function, which is linked to serious health issues, including falls and related injuries, a loss of independence, institutionalization, and mortality (Fried et al. 2001; Cesari et al. 2005). Previous research has shown that weight loss due to insufficient food intake and anorexia increases the risk of malnutrition amongst the older population (Buys et al. 2014; Landi et al. 2017). Diet plays an important role in susceptibility to chronic diseases during the ageing process (Mendoza et al. 2007). Moreover, there are many conditions that can contribute to a decline in appetite or interfere with the ability to eat, which subsequently increases the risk of malnutrition (Schilp et al. 2011; Pilgrim et al. 2016). Physical conditions that may contribute to increasing the risk of malnutrition include decreased saliva production; poor dentition, including difficulties in chewing and wearing dentures (Schilp et al. 2011; Pilgrim et al. 2016); and a reduced sense of taste and poor oral health (Solemdal et al. 2012). Since gastric emptying occurs more slowly in older people, food tends to remain longer in the stomach, prolonging satiation, which can reduce their appetite (de Boer et al. 2013).

Few meta-analyses and reviews on appetite amongst community-dwelling older adults have been conducted (Giezenaar et al. 2016; O’Keeffe et al. 2019). One meta-analysis on appetite reported that older adults have lower levels of appetite and energy intake than do younger adults, suggesting that ageing itself affects food intake (Pilgrim et al. 2016; Giezenaar et al. 2016). It has been reported that older adults with a reduced appetite are more likely to have illnesses and physical conditions that affect appetite (Lee et al. 2006). Other studies have reported that community-dwelling older adults with a poor appetite and those with a good appetite have different levels of dietary intake (Vesnaver et al. 2012; Meij et al. 2017). In general, older adults experience a gradual decline in muscle mass, strength, and function with ageing (Granic et al. 2017). A balanced diet is essential for older adults to live an independent and healthy life (McLean et al. 2016; Rempe et al. 2019). Evidence shows that people with a poor appetite or ability to eat have a significantly lower protein consumption than do controls (Meij et al. 2017). Malnutrition as well as insufficient protein and energy intake are associated with an increased risk of functional decline and mobility limitations (van der Meij et al. 2017; Houston et al. 2017; Rempe et al. 2019). However, few studies have examined the associations of a decreased ability to eat or a poor appetite with physical performance, muscle mass, and muscle strength amongst community-dwelling older adults (Schilp et al. 2011; Chang and Lin 2016). The aim of the current study was to investigate whether having an illness or physical condition affecting one’s appetite or ability to eat is associated with body composition, muscle strength, and physical function amongst community-dwelling older adults.

## Methods

### Age gene/environment susceptibility (AGES)—Reykjavik study

The current study has a cross-sectional design and includes a secondary analysis of data from a large community-based population residing in Reykjavik, Iceland (*n* = 5764). The AGES-Reykjavik Study, which is a part of the Reykjavik study, was established in 1967 to prospectively study cardiovascular disease in Iceland. The AGES-Reykjavik Study is an epidemiological study focused on four biologic systems: the vascular, neurocognitive (including sensory), and musculoskeletal systems and body composition/metabolism (Harris et al. 2007). In brief, a total of 30,795 men and women who were born in 1907–1935 and were living in Reykjavik, Iceland were followed as part of the Reykjavik Study (Sigurdsson et al. 1995). In 2002, the cohort members were re-invited to participate in the AGES-Reykjavik Study. At that time, 11,549 (38%) participants from the Reykjavik Study were still alive. From this group, participants were randomly selected (Saczynski et al. 2008). The AGES-Reykjavik examination for each participant was completed in three clinic visits that took place within four to six weeks. Details of the study design and the baseline AGES-Reykjavik Study assessments have been described elsewhere (Harris et al. 2007).

### Assessment of appetite or the ability to eat

Illnesses or physical conditions affecting appetite or the ability to eat were assessed by a questionnaire during the second clinic visit for the AGES-Reykjavik Study. The first question related to this issue was as follows: “Do you have an illness or physical condition that interferes with your appetite or ability to eat?” (yes/no). For the participants who answered “yes”, a second question was asked, with the following response options: (1) problems with your teeth, (2) swallowing problems, (3) pain when chewing, (4) poor sense of taste, (5) poor sense of smell, (6) stomach/abdominal pain, (7) gas/bloating, (8) unspecific problems with digestion or heartburn, (9) diarrhoea, and (10) other types of illness, including chronic long- and short-term diseases. The status of having a poor appetite or ability to eat was defined on the basis of the first question. The participants who reported having illnesses or physical conditions interfering with their appetite or ability to eat were considered to have a poor appetite, and those who did not report these conditions were considered to have a normal appetite.

### Anthropometric measurements

Body weight was measured whilst the participants were wearing only light undergarments and socks. Each participant stood quietly in the centre of the platform (Marel M1100, Iceland) with his/her weight equally distributed on both feet, without touching any other form of support. Standing height was measured in centimetres (cm) when the participant stood with his/her back against the wall on a wall-mounted stadiometer (Seca stadiometer 242, Germany), with the heels together. Body mass index (BMI) was calculated as the body weight in kg divided by the height in metres squared (kg/m^2^). According to the BMI classification system from the World Health Organization (WHO 2019), the following groups were identified based on BMI: underweight (BMI below 18.5 kg/m^2^), normal (BMI 18.5 to 24.9 kg/m^2^), overweight (BMI 25.0 to 29.9 kg/m^2^) and obese (BMI ≥ 30 kg/m^2^).

Fat-free mass (FFM) and body fat mass (BFM) were assessed using bioelectrical impedance analysis (BIA) (Xitron 4200 Analyser with BIS4200 Utilities Software, San Diego, CA, USA). Whilst Dual-energy X-ray absorptiometry is considered the gold standard measurement tool for body composition in large epidemiological studies, BIA is frequently used to evaluate body composition for both epidemiological and clinical purposes (Buchholz et al. 2004; Marra et al. 2019). Evidence has shown that BIA results are highly correlated with DXA results (Ramel et al. 2011), and BIA is a relatively simple, low cost, quick, and non-invasive technique (Buchholz et al. 2004; Dehghan and Merchant 2008). FFM was calculated on the basis of the sex, height, weight, and resistance of the individuals using the following equation, FFM (kg) = 4.228 + (0.463 * *H*^2^/*R*50) + (0.293 * *W*) + (5.060 * sex), where *H* = body height (cm), *R*50 = resistance 50 kHz, *W* = body weight (kg), and sex = 0 for women and 1 for men. BFM was calculated as follows: BFM (kg) = total weight (kg) − FFM (kg).

### Assessment of muscle strength

#### Knee extension strength

The maximum isometric strength in knee extension on the dominant side was measured using an adjustable and computerized dynamometer on a fixed chair (Good Strength, Metitur, Palokka. Finland). Knee extension strength is considered a standard measure of leg muscle strength (Rantanen et al. 1994, 1999). Knee extension strength was measured in Newtons at a fixed knee angle of 60 degrees from full extension using a strain-gauge transducer that was fastened to the ankle with a belt. Three 4-s trials were performed with 30 s of rest between trials. The highest performance value was used for the analysis (Heistaro and Kansanterveyslaitos 2008). Participants were excluded from the test if they reported having had surgery on their legs or experienced any ischaemic heart conditions within the past 2 months.

#### Grip strength

Grip strength is an indicator of mobility and a determinant of functional performance amongst community-living older adults (Sallinen et al. 2010); it was assessed using an adjustable and computerized dynamometer on a fixed chair, with the participant in a sitting position (Good Strength, Metitur, Palokka. Finland). The chair was equipped with armrests that were height-adjustable to ensure that the participant’s shoulders were relaxed whilst they held the bar positioned directly above the elbow. Three trials were performed with the dominant hand to measure the highest grip strength using the same procedure used for knee extension strength. Each trial lasted 4 s, and after each trial, the participant was allowed to rest for half a minute. The participants were instructed to squeeze the bar using his/her maximum strength. The highest maximum force (N) was used for analysis in the current study (Heistaro and Kansanterveyslaitos 2008). Participants were excluded from the test if they reported having had surgery on their hand or experienced any ischaemic heart conditions within the past 2 months.

### Assessment of physical function

Physical function was measured using the assessments that have been used in other large epidemiological and clinical studies, including the 6-m walk test and the timed up-and-go (TUG) test (Lord et al. 2003). Physical function amongst older adults is generally measured with tests of lower extremity physical performance, including the 6-m walk test and TUG test (Cesari et al. 2005).

### 6-m walk test

The time it took for individuals to walk a distance of 6 m at their usual pace was measured in seconds. The short-distance walk test is reliable when it is performed with standardized methods, and it is easy for older people to complete (Harris et al. 2007). The total walk time was measured two times, and the average of the two trials was used in the study.

### Timed up-and-go test

The TUG test is useful in monitoring clinical changes over time and in evaluating the outcomes of an intervention programme (Whitney et al. 1998). For the TUG test, the total time from when the participant stands up from a sitting position to when the participant has walked 3 m, turned around, walked back to the chair, and sat down again is measured in seconds. The TUG test is well known to be a useful screening tool for older people with balance problems (Podsiadlo and Richardson 1991). The participants were required to use their own footwear and could use a cane or walker if necessary. Participants were excluded if they were not able to rise from the chair (height = 45.5 cm) or walk without assistance. The time for the first completed trial was used in the study.

### Activities of daily living (ADL)

In assessing physical function amongst older adults, epidemiologic studies often count the ADL items that participants find difficult or cannot perform (Jette 1994; Fried et al. 1999; Ostir et al. 2001; Tinetti et al. 2011). In this study, ADL ability was measured by self-reported difficulty in performing five different activities: walking across the room, eating, dressing, bathing, and getting in and out of bed without assistance. In their answers to each ADL question, participants selected between four levels of difficulty (no difficulty, some difficulty, much difficulty, and unable to perform the activity). The response for each ADL item was converted into a dichotomous scale (Lawton and Brody 1969). The choices of some difficulty, much difficulty, and unable to perform the activity were coded as 1. The choice of no difficulty was coded as 0. The summary score of the 5-item ADL questionnaire ranged from 0 to 5, where a score of 0 indicated that the participant had no difficulty performing any of the 5 ADL items, and scores of 1–5 corresponded to the number of ADL items which participants had difficulty in performing or were unable to perform. The reliability and validity of the ADL assessment used in the current study and the data obtained have not been studied.

### Statistical analysis

The first analysis compared the characteristics of two groups (those with a poor appetite and those with a normal appetite). Analysis of variance was used for continuous variables, and chi-square (*χ* ^2^) tests were used for categorical variables. Second, linear regression analysis was performed with various models adjusted for covariates to examine the association of appetite or the ability to eat with body composition, muscle strength, and physical function. Model 1 was adjusted for age and sex, model 2 was additionally adjusted for FFM (or BFM) and height, and model 3 was additionally adjusted for the level of physical activity within the past 12 months and smoking habits. Weekly physical activity (never, rarely, < 1 h, 1–3 h, 4–7 h, > 7 h) and smoking habits (never and previously/current) were assessed by a self-report screening questionnaire constructed for the AGES-Reykjavik Study (Harris et al. 2007; Mijnarends et al. 2016). Model 3 was repeated in the subgroup after excluding people with an underweight BMI. The analyses were separately conducted for the parameters of body composition and physical function. For the association between appetite or the ability to eat and physical function, model 2 was adjusted for BFM because it is a more precise measurement than is weight or BMI (Beaudart et al. 2015). A general linear model was used to examine the trend in the prevalence of a poor appetite in quartiles of the continuous outcome variables and ADL dependence. *p* < 0.05 was considered statistically significant in all analyses, and all statistical analyses were performed using STATA software, version 10 (Statacorp, Texas, USA).

## Results

Amongst the AGES-Reykjavik Study population (*n* = 5764), 804 (14%) individuals reported having illnesses or physical conditions interfering with their appetite or ability to eat and were included in the poor appetite group. Compared with the normal appetite group, the poor appetite group had a lower body weight, height, and FFM; less muscle strength; and worse physical function (Table 1).

**Table 1:** Characteristics of study population according to appetite status (*n* = 5764)

Variable	Total	Normal appetite (*n* = 4960)			Poor appetite (*n* = 804)			Trend *P*	Age agj
	*N*	*N*	*M*	SD	*N*	*M*	SD		*p* value
Age (year)	5764	4960	77.0	5.9	804	77.2	5.8		
Women, *n* (%)	5764	4960	2803	(56.5)	804	523	(66.1)		
Height (cm)	5700	4897	167.0	9	803	165.4	9.4		< 0.0001
Men	2416	2136	175.3	5.8	280	175.1	6.1		0.773
Women	3284	2761	160.7	6.3	523	160.2	6.0		0.155
Weight (kg)	5704	4900	75.8	14.7	804	73.2	14.7		< 0.0001
Men	2417	2136	82.9	13.3	281	80.2	13.8		0.002
Women	3287	2764	70.2	13.2	523	69.5	13.8		0.409
Current BMI (weight in kg/height in m^2^)	5696	4893	27.1	4.4	803	26.7	4.7	0.002	0.064
Underweight, *n* (%)	86		63	(1.3)		23	(2.9)		
Normal, *n* (%)	1837		1535	(32.0)		272	(33.9)		
Overweight, *n* (%)	2502		2176	(44.5)		326	(43.9)		
Obese, *n* (%)	1271		1089	(22.3)		182	(22.7)		
Body fat percent (%)	4069	3521	28.6	(8.2)	548	29.8	(9.1)		0.002
Body fat mass (kg)	4069	3521	21.8	7.8	548	22.0	8.5		0.544
Fat-free mass percent (%)	4069	3521	71.4	(8.2)	548	70.2	(9.1)		0.002
Fat-free mass (kg)	4069	3521	54.0	11.5	548	51.1	10.8		< 0.0001
Fat-free mass index (fat-free mass/height in m^2^)	4069	3521	19.07	2.7	548	18.5	2.7		< 0.0001
Physical activity (h/week)	5299	4533	1.2	2.2	766	1.0	2.0		0.022
Smoking habits, *n* (%)	5569	4765			804			0.468	0.319
Never smoker, *n* (%)	2468		2114	(44.4)		354	(44.0)		
Former smoker *n* (%)	2418		2077	(43.6)		341	(42.4)		
Current smoker, *n* (%)	683		574	(12.1)		109	(13.6)		
Knee extension (N)	5071	4381	322.1	118.9	690	293.2	111.3		< 0.0001
Grip strength (N)	5028	4341	301.4	112.3	687	276.2	103.3		< 0.0001
6-m walk (s)	5350	4605	6.7	2.0	745	7.1	2.1		< 0.0001
Timed up and go (s)	5362	4613	12.5	3.6	749	13.2	4.3		< 0.0001
ADL dependence	5324	4554	0.5	0.9	770	0.7	1.2		< 0.0001

*N*: number of participants, *M*: mean, SD: standard deviation, BMI: body mass index, ADL: activities of daily living

Table 2 shows the association between appetite or the ability to eat and body composition. After the full adjustment in model 3 (age, sex, BFM/FFM, height, PA, and smoking status), compared with the individuals who had no illnesses or conditions interfering with their appetite or ability to eat, those with a poor appetite or ability to eat had a significantly lower BMI (Model 3: beta = − 0.25, 95% confidence interval − 0.40 to − 0.10, *p* = 0.001) and FFM (− 0.73, − 1.15 to − 0.32, *p* = 0.001), whilst the association of a poor appetite or ability to eat with BFM was not significant. After the participants with an underweight BMI were excluded from model 3 for both BMI and FFM, the association was still significant, with 12–16% attenuation from model 3.

**Table 2:** Association between a poor appetite and body composition amongst community-dwelling older adults

Poor appetite versus normal appetite	Total	Poor appetite	95% confidence interval			
	*N*	*N*	Coefficient	Lower	Upper	*p* value
*Body mass index*						
Model 1	5696	803	− 0.34	− 0.67	− 0.01	0.043
Model 2	4069	548	− 0.26	− 0.43	− 0.10	0.002
Model 3	3955	545	− 0.25	− 0.40	− 0.10	0.001
Model 3—excluded underweight BMI	3898	530	− 0.21	− 0.36	− 0.06	0.006
*Fat-free mass*						
Model 1	4069	548	− 1.14	− 1.76	− 0.53	0.000
Model 2	4069	548	− 0.77	− 1.22	− 0.32	0.001
Model 3	3955	545	− 0.73	− 1.15	− 0.32	0.001
Model 3—excluded underweight BMI	3898	530	− 0.60	− 1.02	− 0.19	0.004
Model 1 = adjusted for age and gender, Model 2 = + body fat mass and height, Model 3 = + physical activity and smoking habit						
*Body fat mass*						
Model 1	4069	548	− 0.34	− 0.99	0.31	0.307
Model 2	4069	548	0.40	− 0.11	0.91	0.126
Model 3	3955	545	0.42	− 0.05	0.90	0.081
Model 3—excluded underweight BMI	3898	530	0.39	− 0.08	0.87	0.102

*BMI* Body mass index

Model 1 = adjusted for age and gender, Model 2 =  + fat-free mass and height, Model 3 =  + physical activity and smoking habits

Table 3 shows the association between a poor appetite or ability to eat with physical performance and ADL dependence. For knee extension strength, individuals with a poor appetite or ability to eat had significantly lower knee extension strength after the adjustment of model 2. However, the association became non-significant (model 3, *p* = 0.053) after the model was adjusted for physical activity and smoking habits. Grip strength was significantly associated with appetite or the ability to eat in model 3 (*p* = 0.018), even after individuals with an underweight BMI were excluded (*p* = 0.011), but it was non-significant after the model was adjusted for FFM (*p* = 0.076). Compared with the people who had a normal appetite, the people who reported having illnesses or conditions interfering with appetite or the ability to eat performed significantly worse in the timed 6-m walk test and TUG test and had higher ADL dependence after the full adjustment. The association remained similar even after the model was additionally adjusted for FFM.

**Table 3:** Association between a poor appetite and muscle strength, physical function and ADL dependence amongst community-dwelling older adults

Poor appetite versus normal appetite	Total	Poor Appetite	95% Confidence Interval			
	*N*	*N*	Coefficient	Lower	Upper	*p* value
*Knee extension strength*						
Model 1	5071	690	− 14.06	− 21.01	− 21.01	0.000
Model 2	3813	497	− 8.57	− 16.71	− 0.42	0.039
Model 3	3719	494	− 8.00	− 16.09	0.10	0.053
Model 3 + fat-free mass	3719	494	− 4.82	− 12.70	3.07	0.231
Model 3 − excluded underweight BMI	3667	480	− 7.82	− 16.01	0.38	0.062
*Grip strength*						
Model 1	5028	687	− 10.38	− 16.46	− 4.29	0.001
Model 2	3766	495	− 8.45	− 15.27	− 1.64	0.015
Model 3	3669	492	− 8.25	− 15.08	− 1.42	0.018
Model 3 + fat-free mass	3669	492	− 6.08	− 12.80	0.65	0.076
Model 3 − excluded underweight BMI	3615	478	− 8.95	− 15.88	− 2.03	0.011
*6-m walk*						
Model 1	5350	745	0.25	0.11	0.40	0.001
Model 2	3995	530	0.23	0.07	0.39	0.006
Model 3	3891	527	0.21	0.05	0.38	0.010
Model 3 + fat-free mass	3891	527	0.21	0.05	0.38	0.010
Model 3 − excluded underweight BMI	3836	512	0.23	0.07	0.40	0.006
*Timed up and go*						
Model 1	5362	749	0.59	0.32	0.86	0.000
Model 2	4005	534	0.64	0.34	0.94	0.000
Model 3	3901	531	0.63	0.33	0.93	0.000
Model 3 + fat-free mass	3901	531	0.65	0.35	0.95	0.000
Model 3 − excluded underweight BMI	3845	516	0.64	0.34	0.94	0.000
*ADL dependence*						
Model 1	5324	770	0.25	0.18	0.33	0.000
Model 2	3976	547	0.21	0.13	0.30	0.000
Model 3	3945	544	0.20	0.12	0.29	0.000
Model 3 + fat-free mass	3945	544	0.20	0.12	0.29	0.000
Model 3 − excluded underweight BMI	3888	529	0.22	0.13	0.30	0.000

*ADL* Activities of daily living

Model 1 = adjusted for age and gender, Model 2 =  + body fat mass and height, Model 3 =  + physical activity and smoking habits

**Fig. 1:**
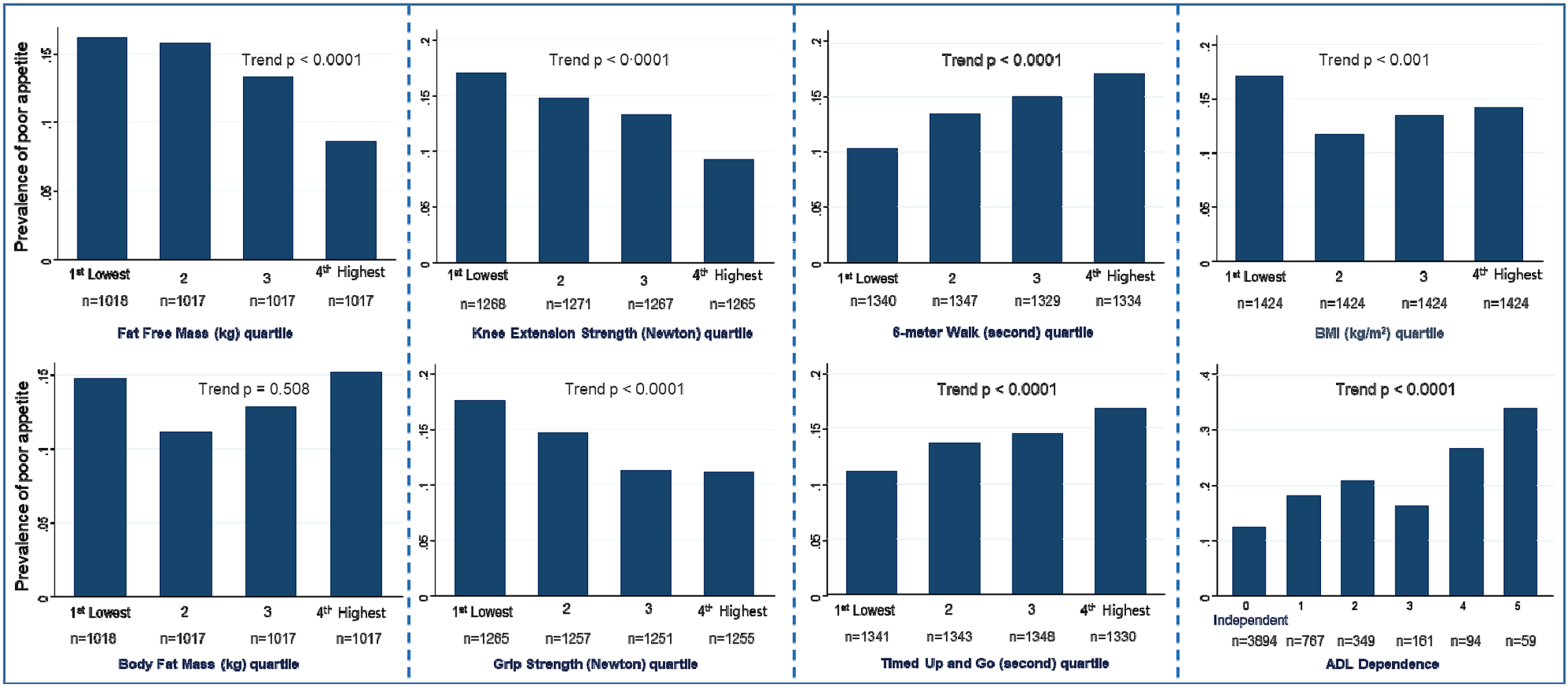
Prevalence of a poor appetite or ability to eat according to the quartile levels* of body composition, muscle strength, physical function, and ADL dependence score amongst the AGES-Reykjavik Study cohort. *Cut-off points for quartile levels of each variable: fat-free mass (kg, < 44.5, 44.6–52.1, 52.2–62.2, 62.2 +), fat mass (kg, < 16.4, 16.5–21.4, 21.5–26.5, 26.5 +), BMI: body mass index (kg/m^2^, < 24.03, 24.03–26.65, 26.66–29.55, 29.56 +), knee extension strength (N, < 231.8, 231.9–300.3, 300.4–390.4, 39.05 +), grip strength (N, < 215.4, 215.8–274.6, 274.7–367.4, 367.9 +), 6-m walk (s, < 5.59, 5.60–6.37, 6.38–7.47, 7.48 +), and timed up and go (s, < 10.24, 10.25–11.76, 11.77–13.90, 13.91 +). ADL dependence (0 = independent, 1–5 = number of ADL items with any difficulty or unable to perform). *ADL* activities of daily living

Figure 1 shows the trend in the unadjusted prevalence of a poor appetite or ability to eat according to the quartile levels of body composition, muscle strength, physical function, and ADL dependence. The prevalence of a poor appetite or ability to eat was the highest in the lowest quartile of BMI, FFM, knee extension strength, and grip strength as well as in the highest quartile/level of the 6-m walk test, TUG test, and ADL dependence scale.

## Discussion

In the current cross-sectional study, we found that having illness or any physical condition interfering with appetite or the ability to eat was associated with body composition and poor physical function in a large cohort of community-dwelling older adults in Iceland, regardless of the participants having a lower BMI or lower BFM. Previous studies have examined the association of malnutrition and poor appetite with various health characteristics (Lee et al. 2006; Reijnierse et al. 2015; Giezenaar et al. 2016). However, research amongst community-dwelling older adults is generally lacking (Beasley et al. 2013; Giezenaar et al. 2016; Houston et al. 2017). Having conditions that contribute to a decline in appetite or interfere with a person’s ability to eat subsequently increases the risk of malnutrition (Schilp et al. 2011). Poor appetite is recognized as a risk factor for low quality of life and functional decline amongst hospitalized patients and nursing home residents (Wilson et al. 2005). For community-dwelling older adults, having a poor appetite can be a predictive factor for the unintentional decline of food intake, which can lead to weight loss (Meij et al. 2017).

The current study has several additional contributions to the current knowledge on the association between appetite or the ability to eat and physical function. The question used to assess appetite in the AGES-Reykjavik Study focused on illnesses or conditions interfering with appetite or the ability to eat, which was different from those used in many previous studies (Wilson et al. 2005; Lee et al. 2006; van der Meij et al. 2017). The association of a poor appetite or ability to eat and muscle strength in the current study provided results similar to those in previous studies (Malafarina et al. 2013; Reijnierse et al. 2015; Lardiés-Sánchez et al. 2017). In particular, grip strength was significantly lower amongst individuals with a poor appetite or ability to eat, whilst knee extension strength showed a trend in the same direction. The strong association between a poor appetite and lower grip strength has also been reported in a previous study (Reijnierse et al. 2015). Our findings suggest that a poor appetite or ability to eat may accelerate functional decline through less muscle strength*.* Previous research has shown that oral pain and chewing impairment in older adults are significantly related to frailty and its components, not only through a nutritional pathway of involuntary weight loss but possibly through a loss of strength and physical performance and reduced physical activity (Okuyama et al. 2011; Kamdem et al. 2017).

Muscle mass explains most of the variance in muscle strength (Visser et al. 2005), indicating that both muscle mass and strength might contribute to a decline in physical function (Visser et al. 2005; Beavers et al. 2013; Reinders et al. 2015). The significant association between appetite or the ability to eat with FFM and muscle strength observed in the current study indicates that individuals having problems interfering with their appetite or ability to eat have lower levels of FFM and muscle strength as well as poorer physical function and higher ADL dependence. Even after additional adjustments were made for BFM and FFM and people with underweight BMI values were excluded from the analysis, having a problem interfering with one’s appetite or ability to eat was strongly associated with poor physical function in the current study. A previous study showed strong evidence that people with a poor appetite, regardless of the cause, are at a high risk of malnutrition (Lardiés-Sánchez et al. 2017). Diet quality may be one of the factors that explain this association because it may vary amongst persons with different appetite levels (van der Meij et al. 2017). The current study suggests there is a link between having problems affecting appetite or the ability to eat and poor physical function amongst community-dwelling older adults, regardless of one’s BMI and fat-free mass. However, future research needs to examine longitudinal associations between appetite and physical function amongst community-dwelling older adults.

Regarding limitations, the current study was a secondary analysis of cross-sectional data. The study used a questionnaire to determine whether the participants had illnesses or condition interfering with their appetite or ability to eat and their current level of physical activity. These questions were originally constructed for screening purposes of the AGES-Reykjavik Study, and the validity of the questions was not studied. Although the information on the validity and reliability of the ADL summary score is not available as part of the current study, it is common practice to present results of ADL assessment by the number of activities that participants reported having difficulty in performing or were unable to perform (Fried et al. 1999; Ostir et al. 2001; Volpato et al. 2008; Tinetti et al. 2011), whilst other studies use scores with various levels of difficulty or inability to perform the activity (Volpato et al. 2007; Iwarsson et al. 2009; Bouwstra et al. 2019). It should be noted that the ADL summary score does not refer to the level of difficulty or disability because the ADL assessment in the current study was different from a conventional ADL assessment. Furthermore, the questionnaire used to assess appetite or the ability to eat was different from the scale used in previous studies, which directly asked participants about their level of appetite (van der Meij et al. 2017). In addition, it is possible that the participants who completed physical performance measurements may have been healthier than those who were excluded or did not complete the measurement. Our analyses did not account for the effect of recent weight loss, the presence of other diseases, or the use of medication, which is nevertheless a major limitation of the study. Finally, due to the cross-sectional study design, it is not possible to draw conclusions about cause and effect relationships because low physical functioning also negatively impacts appetite or the ability to eat amongst community-dwelling older adults (Lee et al. 2006).

In conclusion, our study shows there is an association between a poor appetite or ability to eat and body composition, muscle strength, and physical function amongst community-dwelling older adults. Our findings have the potential to encourage both older individuals and the community to be aware of conditions that can interfere with or decrease appetite or the ability to eat, which could prevent negative consequences, such as malnutrition. Although some of the illnesses or conditions reported to interfere with appetite or the ability to eat are seldom considered to be severe (such as heartburn, gas, or bloating), their strong associations with physical function suggest that any condition interfering with appetite or the ability to eat amongst community-dwelling older adults requires attention.

## References

[CR1] Beasley JM, Wertheim BC, LaCroix AZ, Prentice RL, Neuhouser ML, Tinker LF, Kritchevsky S, Shikany JM, Eaton C, Chen Z, Thomson CA (2013). Biomarker-calibrated protein intake and physical function in the women’s health initiative. J Am Geriatr Soc.

[CR57] Beaudart C, Reginster JY, Slomian J, Buckinx F, Dardenne N, Quabron A, Slangen C, Gillain S, Petermans J, Bruyère O (2015). Estimation of sarcopenia prevalence using various assessment tools. Experimental Gerontology.

[CR2] Beavers KM, Beavers DP, Houston DK, Harris TB, Hue TF, Koster A, Newman AB, Simonsick EM, Studenski SA, Nicklas BJ, Kritchevsky SB (2013). Associations between body composition and gait-speed decline: results from the health, aging, and body composition study 1234. Am J Clin Nutr.

[CR3] Bouwstra H, Smit EB, Wattel EM, van der Wouden JC, Hertogh CMPM, Terluin B, Terwee CB (2019). Measurement properties of the Barthel index in geriatric rehabilitation. J Am Med Dir Assoc.

[CR4] Buchholz AC, Bartok C, Schoeller DA (2004). The validity of bioelectrical impedance models in clinical populations. Nutr Clin Pract.

[CR5] Buys DR, Roth DL, Ritchie CS, Sawyer P, Allman RM, Funkhouser EM, Hovater M, Locher JL (2014). Nutritional risk and body mass index predict hospitalization, nursing home admissions, and mortality in community-dwelling older adults: results from the UAB study of aging with 8.5 years of follow-up. J Gerontol A Biol Sci Med Sci.

[CR6] Cesari M, Kritchevsky SB, Penninx BWHJ, Nicklas BJ, Simonsick EM, Newman AB, Tylavsky FA, Brach JS, Satterfield S, Bauer DC, Visser M, Rubin SM, Harris TB, Pahor M (2005). Prognostic value of usual gait speed in well-functioning older people—results from the health, aging and body composition study. J Am Geriatr Soc.

[CR7] Chang S-F, Lin P-L (2016). Prefrailty in community-dwelling older adults is associated with nutrition status. J Clin Nurs.

[CR8] de Boer A, Ter Horst GJ, Lorist MM (2013). Physiological and psychosocial age-related changes associated with reduced food intake in older persons. Ageing Res Rev.

[CR9] Dehghan M, Merchant AT (2008). Is bioelectrical impedance accurate for use in large epidemiological studies?. Nutr J.

[CR10] Fried LP, Bandeen-Roche K, Kasper JD, Guralnik JM (1999). Association of comorbidity with disability in older women: the women’s health and aging study. J Clin Epidemiol.

[CR11] Fried LP, Tangen CM, Walston J, Newman AB, Hirsch C, Gottdiener J, Seeman T, Tracy R, Kop WJ, Burke G, McBurnie MA (2001). Frailty in older adults evidence for a phenotype. J Gerontol A Biol Sci Med Sci.

[CR12] Giezenaar C, Chapman I, Luscombe-Marsh N, Feinle-Bisset C, Horowitz M, Soenen S (2016). Ageing is associated with decreases in appetite and energy intake—a meta-analysis in healthy adults. Nutrients.

[CR13] Granic A, Mendonça N, Sayer AA, Hill TR, Davies K, Adamson A, Siervo M, Mathers JC, Jagger C (2017). Low protein intake, muscle strength and physical performance in the very old: the Newcastle 85+ study. Clin Nutr.

[CR14] Harris TB, Launer LJ, Eiriksdottir G, Kjartansson O, Jonsson PV, Sigurdsson G, Thorgeirsson G, Aspelund T, Garcia ME, Cotch MF, Hoffman HJ, Gudnason V (2007). Age, gene/environment susceptibility-reykjavik study: multidisciplinary applied phenomics. Am J Epidemiol.

[CR15] Heistaro S, Kansanterveyslaitos (2008). Methodology report: health 2000 Survey.

[CR17] Houston DK, Tooze JA, Garcia K, Visser M, Rubin S, Harris TB, Newman AB, Kritchevsky SB, The Health ABC Study (2017). Protein intake and mobility limitation in community-dwelling older adults: the health ABC study. J Am Geriatr Soc.

[CR18] Iwarsson S, Horstmann V, Sonn U (2009). Assessment of dependence in daily activities combined with a self-rating of difficulty. J Rehabil Med.

[CR19] Jette AM (1994). How measurement techniques influence estimates of disability in older populations. Soc Sci Med.

[CR20] Kamdem B, Seematter-Bagnoud L, Botrugno F, Santos-Eggimann B (2017). Relationship between oral health and Fried’s frailty criteria in community-dwelling older persons. BMC Geriatr.

[CR22] Landi F, Calvani R, Tosato M, Martone AM, Picca A, Ortolani E, Savera G, Salini S, Ramaschi M, Bernabei R, Marzetti E (2017). Animal-derived protein consumption is associated with muscle mass and strength in community-dwellers: results from the Milan Expo survey. J Nutr Health Aging.

[CR23] Lardiés-Sánchez B, Sanz-París A, Pérez-Nogueras J, Serrano-Oliver A, Torres-Anoro ME, Cruz-Jentoft AJ (2017). Influence of nutritional status in the diagnosis of sarcopenia in nursing home residents. Nutrition.

[CR24] Lawton MP, Brody EM (1969). Assessment of older people: self-maintaining and instrumental activities of daily living. Gerontologist.

[CR25] Lee JS, Kritchevsky SB, Tylavsky F, Harris TB, Ayonayon HN, Newman AB (2006). Factors associated with impaired appetite in well-functioning community-dwelling older adults. J Nutr Elder.

[CR58] Lord SR, Menz HB, Tiedemann A (2003). A physiological profile approach to falls risk assessment and prevention. Phys Ther.

[CR26] Malafarina V, Uriz-Otano F, Gil-Guerrero L, Iniesta R (2013). The anorexia of ageing: physiopathology, prevalence, associated comorbidity and mortality. A systematic review. Maturitas.

[CR27] Marra M, Sammarco R, De Lorenzo A, Iellamo F, Siervo M, Pietrobelli A, Donini LM, Santarpia L, Cataldi M, Pasanisi F, Contaldo F (2019). Assessment of body composition in health and disease using bioelectrical impedance analysis (BIA) and dual energy X-ray absorptiometry (DXA): a critical overview. Contrast Media Mol Imaging.

[CR28] McLean RR, Mangano KM, Hannan MT, Kiel DP, Sahni S (2016). Dietary protein intake is protective against loss of grip strength among older adults in the Framingham offspring cohort. J Gerontol A Biol Sci Med Sci.

[CR29] Mendoza JA, Drewnowski A, Christakis DA (2007). Dietary energy density is associated with obesity and the metabolic syndrome in U.S. adults. Diabetes Care.

[CR56] Mijnarends DM, Koster A, Schols JMGA, Meijers JMM, Halfens RJG, Gudnason V, Eiriksdottir G, Siggeirsdottir K, Sigurdsson S, Jónsson PV, Meirelles O, Harris T (2016). Physical activity and incidence of sarcopenia: the population-based AGES—Reykjavik Study. Age Ageing.

[CR30] O’Keeffe M, Kelly M, O’Herlihy E, O’Toole PW, Kearney PM, Timmons S, O’Shea E, Stanton C, Hickson M, Rolland Y, Sulmont Rossé C, Issanchou S, Maitre I, Stelmach-Mardas M, Nagel G, Flechtner-Mors M, Wolters M, Hebestreit A, De Groot LCPGM, van de Rest O, Teh R, Peyron MA, Dardevet D, Papet I, Schindler K, Streicher M, Torbahn G, Kiesswetter E, Visser M, Volkert D, O’Connor EM (2019). Potentially modifiable determinants of malnutrition in older adults: a systematic review. Clin Nutr.

[CR31] Okuyama N, Yamaga T, Yoshihara A, Nohno K, Yoshitake Y, Kimura Y, Shimada M, Nakagawa N, Nishimuta M, Ohashi M, Miyazaki H (2011). Influence of dental occlusion on physical fitness decline in a healthy Japanese elderly population. Arch Gerontol Geriatr.

[CR32] Ostir GV, Volpato S, Kasper JD, Ferrucci L, Guralnik JM (2001). Summarizing amount of difficulty in ADLs: a refined characterization of disability. Results from the women’s health and aging study. Aging (Milano).

[CR33] Pilgrim AL, Baylis D, Jameson KA, Cooper C, Sayer AA, Robinson SM, Roberts HC (2016). Measuring appetite with the simplified nutritional appetite questionnaire identifies hospitalised older people at risk of worse health outcomes. J Nutr Health Aging.

[CR34] Podsiadlo D, Richardson S (1991). The Timed “up & go”: a test of basic functional mobility for frail elderly persons. J Am Geriatr Soc.

[CR35] Ramel A, Geirsdottir OG, Arnarson A, Thorsdottir I (2011). Regional and total body bioelectrical impedance analysis compared with DXA in Icelandic elderly. Eur J Clin Nutr.

[CR36] Rantanen T, Era P, Heikkinen E (1994). Maximal isometric strength and mobility among 75-year-old men and women. Age Ageing.

[CR37] Rantanen T, Guralnik JM, Ferrucci L, Leveille S, Fried LP (1999). Coimpairments: strength and balance as predictors of severe walking disability. J Gerontol A Biol Sci Med Sci.

[CR38] Reijnierse EM, Trappenburg MC, Leter MJ, Blauw GJ, de van der Schueren MAE, Meskers CGM, Maier AB (2015). The association between parameters of malnutrition and diagnostic measures of Sarcopenia in geriatric outpatients. PLoS ONE.

[CR39] Reinders I, Murphy RA, Koster A, Brouwer IA, Visser M, Garcia ME, Launer LJ, Siggeirsdottir K, Eiriksdottir G, Jonsson PV, Gudnason V, Harris TB (2015). Muscle quality and muscle fat infiltration in relation to incident mobility disability and gait speed decline: the age, gene/environment susceptibility-Reykjavik study. J Gerontol A Biol Sci Med Sci.

[CR40] Rempe HM, Sproesser G, Gingrich A, Spiegel A, Skurk T, Brandl B, Hauner H, Renner B, Volkert D, Sieber CC, Freiberger E, Kiesswetter E (2019). Measuring eating motives in older adults with and without functional impairments with The Eating Motivation Survey (TEMS). Appetite.

[CR42] Saczynski JS, Jónsdóttir MK, Garcia ME, Jonsson PV, Peila R, Eiriksdottir G, Olafsdottir E, Harris TB, Gudnason V, Launer LJ (2008). Cognitive impairment: an increasingly important complication of type 2 diabetes. Am J Epidemiol.

[CR43] Sallinen J, Stenholm S, Rantanen T, Heliövaara M, Sainio P, Koskinen S (2010). Hand-grip strength cut-points to screen older persons at risk for mobility limitation. J Am Geriatr Soc.

[CR44] Schilp J, Wijnhoven HAH, Deeg DJH, Visser M (2011). Early determinants for the development of undernutrition in an older general population: longitudinal aging study Amsterdam. Br J Nutr.

[CR45] Sigurdsson E, Thorgeirsson G, Sigvaldason H, Sigfusson N (1995). Unrecognized myocardial infarction: epidemiology, clinical characteristics, and the prognostic role of angina pectoris. The Reykjavik Study. Ann Intern Med.

[CR46] Solemdal K, Sandvik L, Willumsen T, Mowe M, Hummel T (2012). The impact of oral health on taste ability in acutely hospitalized elderly. PLoS ONE.

[CR47] Tinetti ME, McAvay G, Chang SS, Ning Y, Newman AB, Fitzpatrick A, Fried TR, Harris TB, Nevitt MC, Satterfield S, Yaffe K, Peduzzi P (2011). Effect of chronic disease-related symptoms and impairments on universal health outcomes in older adults. J Am Geriatr Soc.

[CR48] van der Meij BS, Wijnhoven HAH, Lee JS, Houston DK, Hue T, Harris TB, Kritchevsky SB, Newman AB, Visser M (2017). Poor appetite and dietary intake in community-dwelling older adults. J Am Geriatr Soc.

[CR49] Vesnaver E, Keller HH, Payette H, Shatenstein B (2012). Dietary resilience as described by older community-dwelling adults from the NuAge study “if there is a will—there is a way!”. Appetite.

[CR50] Visser M, Goodpaster BH, Kritchevsky SB, Newman AB, Nevitt M, Rubin SM, Simonsick EM, Harris TB (2005). Muscle mass, muscle strength, and muscle fat infiltration as predictors of incident mobility limitations in well-functioning older persons. J Gerontol A Biol Sci Med Sci.

[CR51] Volpato S, Onder G, Cavalieri M, Guerra G, Sioulis F, Maraldi C, Zuliani G, Fellin R (2007). Characteristics of nondisabled older patients developing new disability associated with medical illnesses and hospitalization. J Gen Intern Med.

[CR52] Volpato S, Cavalieri M, Guerra G, Sioulis F, Ranzini M, Maraldi C, Fellin R, Guralnik JM (2008). Performance-based functional assessment in older hospitalized patients: feasibility and clinical correlates. J Gerontol A Biol Sci Med Sci.

[CR53] Whitney SL, Poole JL, Cass SP (1998). A review of balance instruments for older adults. Am J Occup Ther.

[CR54] WHO (2019) Body mass index—BMI. https://www.euro.who.int/en/health-topics/disease-prevention/nutrition/a-healthy-lifestyle/body-mass-index-bmi. Accessed 17 Dec 2019

[CR55] Wilson M-MG, Thomas DR, Rubenstein LZ, Chibnall JT, Anderson S, Baxi A, Diebold MR, Morley JE (2005). Appetite assessment: simple appetite questionnaire predicts weight loss in community-dwelling adults and nursing home residents. Am J Clin Nutr.

